# Prenatal Diagnosis of Isolated Right Aortic Arch: A Collaborative Approach to Management of Postnatal Aerodigestive Symptoms

**DOI:** 10.1002/ppul.71381

**Published:** 2025-11-14

**Authors:** Jonathan N. Flyer, Kelly J. Knight, Erika M. Edwards, Caitlin S. Haxel, Thomas Lahiri

**Affiliations:** ^1^ Department of Pediatrics University of Vermont Burlington Vermont USA; ^2^ Robert Larner MD College of Medicine University of Vermont Burlington Vermont USA; ^3^ Division of Pediatric Cardiology The University of Vermont Children's Hospital Burlington Vermont USA; ^4^ Vermont Oxford Network Burlington Vermont USA; ^5^ Department of Mathematics and Statistics College of Engineering and Mathematical Sciences Burlington Vermont USA; ^6^ Division of Pediatric Pulmonology The University of Vermont Children's Hospital Burlington Vermont USA

**Keywords:** care pathway, pediatric cardiology, pediatric pulmonology, vascular ring

## Abstract

**Background:**

Although prenatal imaging advancements have increased isolated right aortic arch (iRAA) detection, optimal postnatal vascular ring management remains uncertain. Whereas early postnatal imaging and surgical management may be clinically warranted for some infants, long‐term data are not yet available to justify an identical approach for all prenatal iRAA diagnoses.

**Methods:**

Children with prenatally diagnosed iRAA and left ductus arteriosus were included in a retrospective cohort study at a rural academic center. Fetal echocardiograms (2014–2024) were reviewed to exclude complex congenital heart disease. Joint postnatal cardiopulmonary care was standardized. Clinical data were ascertained using aerodigestive symptom checklists. Fisher's exact test was used to examine associations between symptoms and surgical referral. Growth was assessed by z‐score rates of change from birth weight until last clinic weight.

**Results:**

3,458 fetal echocardiograms were performed yielding 31 distinct cases meeting inclusion criteria, with postnatal data available for 25. Twenty were managed medically (25% asymptomatic, 75% intermittently symptomatic) with median follow up of 14 (11.5, 17.5) months; five underwent surgery (80% progressively symptomatic) with median follow up of 36 (18, 37) months). Choking/dysphagia (*p* = 0.04), recurrent emesis (*p* = 0.03), and the combination of both digestive symptoms and noisy breathing (*p* = 0.01) were associated with surgical referral. Weight outcomes were similar (*p* = 0.98) between medical and surgical patients.

**Conclusions:**

Although aerodigestive symptoms were common, most were not progressive and weight profiles were not adversely affected. Isolated digestive symptoms or in combination with respiratory symptoms may prompt greater concern. A collaborative cardiopulmonary approach to clinical evaluation may help with short‐term risk stratification.

## 1 Introduction

Prenatal imaging standards have evolved over the past decade to include anatomic evaluation of aortic arch sidedness, increasing the fetal diagnosis of isolated right aortic arch (iRAA) [1, 2, 3, 4]. In 2013 the International Society of Ultrasound in Obstetrics and Gynecology recommended including the three‐vessel trachea view (3VTV) during the 20‐week anatomy scan, further supported in 2018 by national practice guidelines [5, 6]. Our institution began including the 3VTV in routine prenatal ultrasounds in 2014, which subsequently increased iRAA fetal diagnostic rates and prompted questions about postnatal management.

Anatomic variants of iRAA with left ductus arteriosus may serve as substrate for a vascular ring by encircling the trachea and esophagus [7]. Although the developing fetus is asymptomatic, postnatally some infants may present with progressive aerodigestive symptoms, often during the first few months of life [8]. Symptoms due to tracheoesophageal compression may require additional clinical investigation, testing, and multidisciplinary care [7, 8]. While more progressive and severe symptoms likely necessitate vascular ring surgery, some centers may still recommend empiric testing and surgical management for all prenatally diagnosed children, even those with absent or milder symptoms [3, 7, 8, 9, 10, 11, 12, 13]. Although current era studies frequently report operative outcomes, much less is known regarding the clinical course of prenatally diagnosed iRAA infants in rural areas who are managed more conservatively [14, 15, 16, 17, 18].

The purpose of this study was to evaluate postnatal aerodigestive symptoms in infants and toddlers with primary fetal diagnosis of iRAA. We focused on the clinical course and short‐term outcomes of patients managed by a collaborative institutional care pathway. The primary aim was to determine the percentage of prenatally diagnosed iRAA patients who were asymptomatic without surgical intervention by 18 months of age. Additionally, we sought to categorize aerodigestive symptom patterns, assess patient growth, and share our collaborative care approach.

## 2 Methods

### 2.1 Setting

This was a single center retrospective cohort study conducted at a rural academic medical center (The University of Vermont Children's Hospital). Our center provides the only maternal fetal medicine, fetal cardiology, pediatric cardiology, and pediatric pulmonology services in the state of Vermont, and extends rural subspecialty care to northeastern New York and the northwest border of New Hampshire. The STROBE checklist for cohort studies was followed (Appendix 1) [19]. All research activities were approved by the University of Vermont Institutional Review Board.

### 2.2 Participant Selection

The study period included ten years (1/01/2014‐12/31/2024) after institutional implementation of the 3VTV. All fetal cardiology referrals during the study period were eligible for inclusion. An institutional fetal echocardiographic database was retrospectively queried for common free text anatomic descriptors of right aortic arches (“right aortic arch,” “right sided aortic arch,” “right‐sided aortic arch,” “right aorta”). Fetal echocardiographic reports and images were reviewed to verify anatomic characteristics and confirm eligibility (JF, CH). Pregnancies with any of the following were excluded: left aortic arch, double aortic arch, right or absent ductus arteriosus, fetal arrhythmias requiring medical therapy, and/or critical congenital heart disease. Fetal echocardiograms that were normal but ordered due to family history of right aortic arch or question of arch sidedness were excluded. Distinct pregnancies with fetal echocardiographic diagnosis of iRAA were then cross‐referenced with a prospective pediatric cardiology quality improvement patient database to assist with maternal‐infant linkage to postnatal outcomes (i.e., eligible pediatric patients).

All eligible pediatric patients were retrospectively reviewed at the conclusion of the study period. Demographic and clinical data were extracted from the electronic health record and included as available or until discharge from the collaborative care pathway. Patients without documented postnatal pediatric pulmonology or cardiology clinic visits were excluded from analysis.

### 2.3 Collaborative Care Pathway

**Figure 1:**
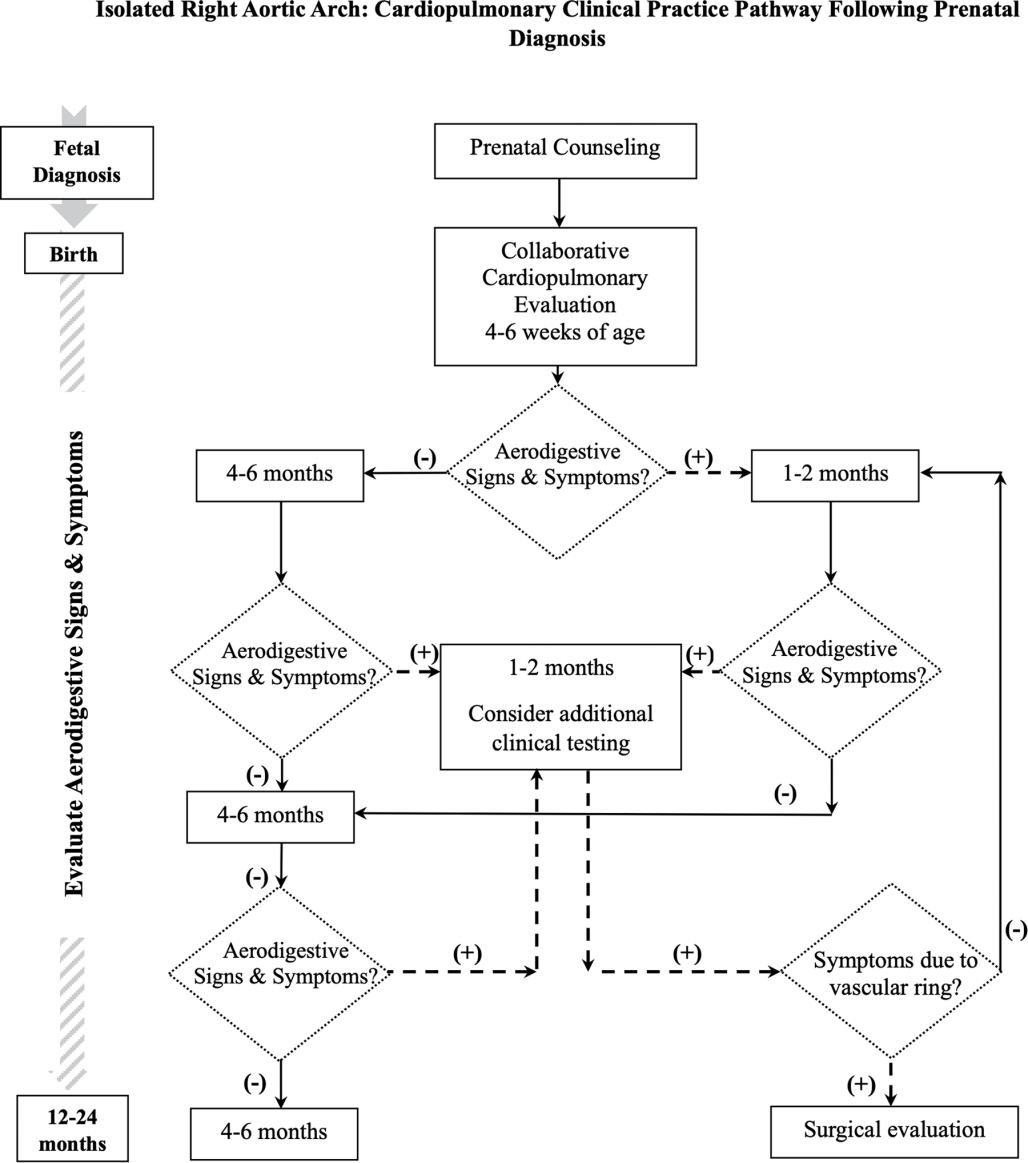
Collaborative cardiopulmonary care pathway for isolated right aortic arch diagnoses confirmed by fetal echocardiogram. Postnatal clinical evaluation frequency is determined by presence (+) or absence (–) of aerodigestive signs and/or symptoms. Additional clinical testing is based on the primary aerodigestive symptom(s), with surgical evaluation considered for progressive findings attributed to the vascular ring anatomy.

Given the uncertainty regarding optimal care for this group of patients, variable clinical approach, and absence of evidence‐based guidelines, a new institutional collaborative care pathway was developed in 2016 by our pediatric cardiology and pulmonology teams (Figure 1) to standardize pre and postnatal care follow up, reduce testing variability, and improve communication with patients and families, their medical home, and subspecialists. The clinical pathway focused on iRAA care following prenatal diagnosis confirmed by fetal echocardiogram and offered initial joint postnatal evaluation by both services. A transthoracic echocardiogram was completed at the first postnatal visit to confirm cardiac anatomy. Clinical follow up and testing was primarily determined by the presence of aerodigestive signs and/or symptoms.

Evaluations focused on both the context and evolution of symptoms including association with upper respiratory infections, presence at baseline, during sleep and/or with feeding, and progression upon advancement to solid foods. All patients were monitored for growth, development, advancing diet, and respiratory symptoms associated with positional changes. An esophagram was considered to evaluate primary feeding symptoms. Contrast chest computed tomography and bronchoscopy were considered for primary respiratory symptoms. Surgical evaluation was considered if symptom progression was primarily attributed to the vascular anatomy. Asymptomatic or minimally symptomatic patients were offered anticipatory guidance and shared decision making to support additional follow up either by the pulmonary service or primary medical home.

### 2.4 Variables and Statistical Methods

Pediatric pulmonologists utilized our institutional care pathway including an objective aerodigestive symptom checklist (Table 1) to standardize analysis. Individual symptoms were evaluated at each clinical visit and a binary approach to the presence/absence of respiratory symptoms or feeding symptoms was taken to minimize subjectivity when grading clinical severity. For the purposes of this study, infants were categorized as asymptomatic or symptomatic determined by respiratory and/or feeding related symptoms. Infant symptoms were then also classified as intermittent or progressive. Concerning symptoms included noisy breathing (including wheezing, stridor, stertor, or raspy/rattly breath sounds) and dysphagia. Isolated noisy breathing was followed medically if dynamic airway collapse (tracheomalacia) was suspected by history. Infants with persistence or progression of symptoms underwent further evaluation to assess the degree of esophageal or tracheal compression.

**Table 1:** Aerodigestive symptom checklist.

	Present	Absent
**Respiratory symptom**		
Wheezing		
Stridor		
Stertor		
Raspy/Rattly breath sounds		
Cough		
Pulmonary infections requiring antibiotics		
Apnea		
Cyanosis		
Preference for upright or prone positioning		
**Feeding‐related Symptom**		
Choking or Dysphagia		
Recurrent emesis		

*Note:* Clinician administered aerodigestive symptom checklist during postnatal follow‐up of isolated right aortic arch patients following prenatal diagnosis.

Demographic and clinical characteristics were described with descriptive statistics including means and medians as appropriate. Analyses were performed utilizing SAS 9.4. Fisher's exact test was used to examine associations between symptoms and surgical referral. Longitudinal growth was assessed by z‐score change from birth weight until weight at last medical evaluation or surgery and tested using a paired *t*‐test. Testing review included echocardiographic, radiographic (contrast chest computed tomography or magnetic resonance angiography), procedural (bronchoscopy), and genetic results.

## 3 Results

**Figure 2:**
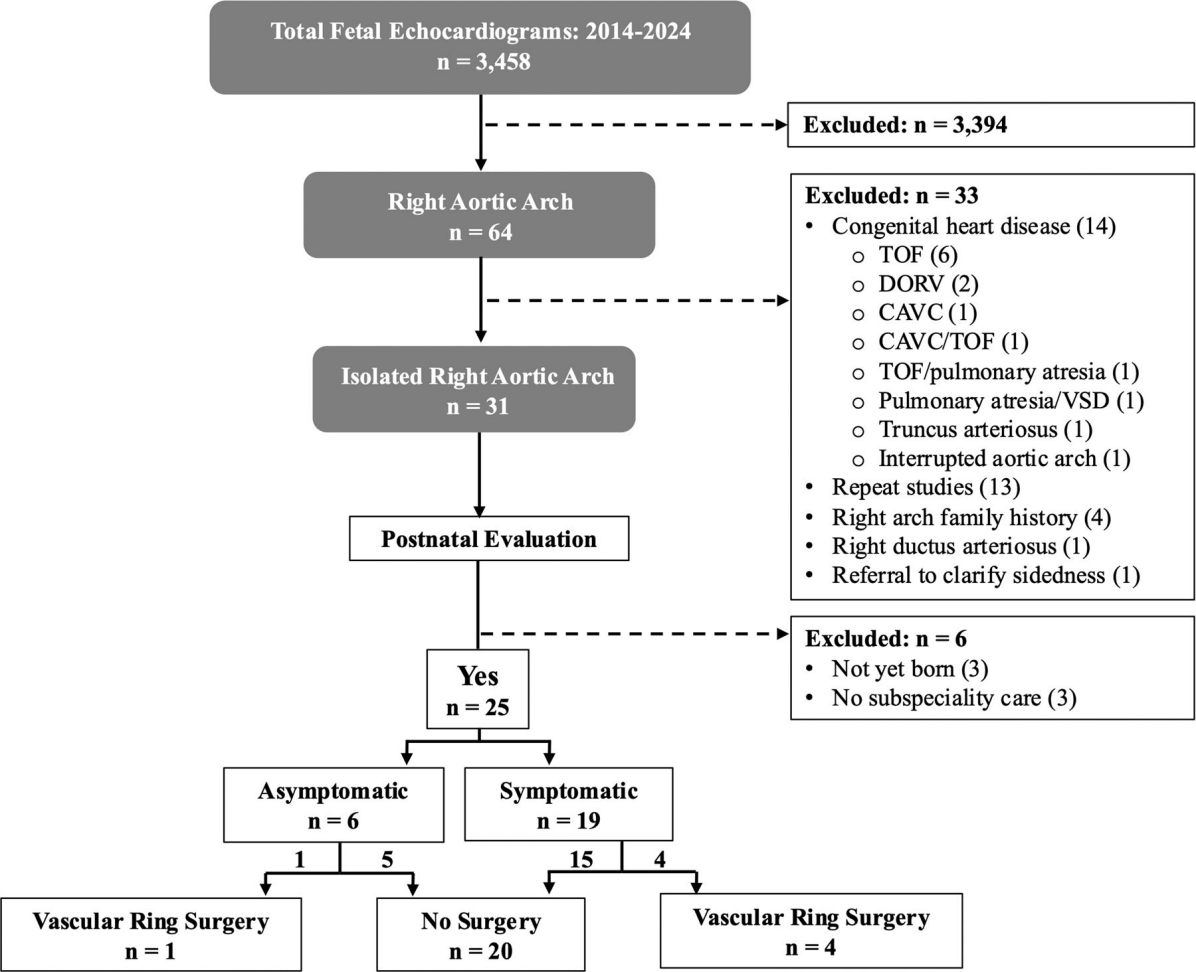
Study inclusion. The flow diagram depicts patient selection and short‐term outcomes. CAVC, complete atrioventricular canal; DORV, double outlet right ventricle; TOF, tetralogy of Fallot; VSD, ventricular septal defect.

A total of 3,458 fetal echocardiograms were performed on 2,444 distinct patients during the study period (Figure 2). Sixty‐four right arch fetal cases were identified, and 31 met iRAA inclusion criteria. Cases were excluded for complex congenital heart disease (14), repeat studies (13), sidedness clarified to be normal (5), and a right sided ductus arteriosus (1). At the conclusion of the study period, three patients were not yet born, and three did not have any postnatal subspeciality evaluation.

A final study cohort of 25 iRAA infants were included for analysis (Table 2). Twenty patients (80%) were medically managed and 5 (20%) were referred for surgery. Median birth weight for medically managed patients was 3,129 grams (including two premature infants) and 3,430 grams for patients referred for surgery. APGARs were normal for both groups. Infants were initially evaluated at median ages of 6 weeks (medical management) or 7 weeks (surgical). First additional testing due to symptoms occurred at a median age of 7.5 months (medical) or 8 months (surgical). Median study follow‐up time was 14 (11.5, 17.5) months in the medical group and 36 (18, 37) months in the surgical group.

**Table 2:** Patient characteristics following prenatal diagnosis of isolated right aortic arch.

Patient characteristics	Management	
	Medical (*n* = 20)	Surgical (*n* = 5)
**Growth**		
1‐min APGAR, median (Q1, Q3)	8 (7, 8)	8 (7, 8)^a^
5‐min APGAR, median (Q1, Q3)	9 (9, 9)	9 (9, 9)^a^
Birth weight in grams, median (Q1, Q3)	3129 (2784, 3475)	3430 (3260, 3680)
Change in weight z‐score, mean (SD)	0.69 (1.91)	0.66 (1.68)
Age at newborn visit in weeks, median (Q1, Q3)	6 (4, 8)	7 (4, 7)
Age at last subspecialty visit (months), median (Q1, Q3)	14 (11.5, 17.5)	36 (18, 37)
**Aerodigestive symptom profiles**		
Asymptomatic	5 (25%)	1 (20%)^b^
Symptomatic	15 (75%)	4 (80%)
**Aerodigestive symptoms**		
Respiratory symptoms	14 (70%)	4 (80%)
Noisy breathing^c^	13 (65%)	4 (80%)
Cough	5 (25%)	2 (40%)
Pulmonary infections requiring antibiotics	2 (10%)	1 (20%)
Apnea	1 (5%)	0 (0%)
Cyanosis	0 (0%)	0 (0%)
Feeding‐related symptoms	7 (35%)	4 (80%)
Choking or dysphagia	5 (25%)	4 (80%)
Recurrent emesis	2 (10%)	3 (60%)
**Anatomy and other medical conditions**		
Arch Descriptors		
Aberrant left subclavian	11 (55%)	3 (60%)
Mirror image branching	9 (45%)	2 (40%)^d^
Other congenital heart disease		
Atrial septal defect	1 (5%)	0 (0%)
Ventricular septal defect	1 (5%)	0 (0%)
Anomalous coronary origin	0 (0%)	1 (20%)
Pulmonary artery hypoplasia	1 (5%)	0 (0%)
Peripheral pulmonary stenosis	2 (10%)	1 (20%)
Prematurity^e^	2 (10%)	0 (0%)
Gastroesophageal reflux	2 (10%)	0 (0%)
Other pulmonary disease		
Asthma	0 (0%)	1 (20%)
Obstructive sleep apnea	1 (5%)	0 (0%)
Other congenital anomaly		
Facial anomalies	1 (5%)	0 (0%)
Horseshoe kidney	1 (5%)	0 (0%)
Polydactyly	1 (5%)	0 (0%)
Prenatal genetic testing		
Noninvasive prenatal testing	17 (85%)	3 (60%)
FISH	2 (10%)	0 (0%)
Microarray	3 (15%)	0 (0%)
Postnatal genetic testing		
FISH	2 (10%)	0 (0%)
Microarray	1 (5%)	0 (0%)
Whole exome sequencing	2 (10%)	0 (0%)
Mitochondrial analysis	1 (5%)	0 (0%)
**Testing**		
Studies performed^f^		
Bronchoscopy	1 (5%)	4 (80%)
Chest computed tomography	0 (0%)	4 (80%)
Esophagram	2 (10%)	4 (80%)
Median age at first additional testing^g^	7.5^h^	8 (2, 10)
Median age at surgical repair, months	—	12 (11, 13)

^a^ *n* = 3.

^b^ This patient received care before establishment of collaborative pathway and was referred for surgery due to suspicion of double aortic arch (later found to be iRAA)

^c^ Includes wheezing and/or raspy/rattly breath sounds; no patients presented with stridor or stertor.

^d^ For one patient, the fetal and postnatal echocardiographic diagnosis was mirror image branching, however, CT demonstrated a double aortic arch (right dominant, diminutive left).

^e^ Defined as < 37 weeks gestation.

^f^ Includes studies performed at our facility.

^g^ Includes computed tomography, magnetic resonance angiography, bronchoscopy, and esophagram; performed at either our facility or referral center.

^h^ *n* = 2.

Of the entire cohort, 13 (52%) total children were asymptomatic by 18 months without surgery. Within the medical group, five patients (25%) remained entirely asymptomatic throughout their postnatal clinical observation while 15 patients (75%) experienced at least one aerodigestive symptom. Of these 15 patients, 10 (66%) became asymptomatic: 8 patients became asymptomatic by 15 months, and an additional two patients became consistently asymptomatic by 24 months. At the end of the study period, aerodigestive symptoms for five patients were either attributed to causes other than a vascular ring or determined to be too mild to warrant surgical referral.

Of the entire cohort, symptoms in six (24%) infants were progressive or severe enough to warrant further investigations with radiographic studies and/or bronchoscopy. Two symptomatic infants (8%) had upper airway or digestive difficulties that were determined to be unrelated to their vascular anatomy. Four infants (16%) were referred for surgery based on severity of symptoms and anatomical factors, (e.g. severe tracheal or esophageal narrowing), that correlated with additional testing, with median age at surgery of 12 months. One child (4%) had surgery due to anatomic findings by CT scan at 2 months of age before any symptom development; this clinical decision was determined to be based on a physician practice preference and was made before the development of the clinical care pathway. Therefore, of the entire prenatal cohort of 25 patients, five (20%) were determined to be asymptomatic and without surgical intervention following prenatal diagnosis through 18 months. An additional 15 patients (60%) were medically managed through mild symptoms, typically intermittent, resolving, and/or attributable to other primary causes.

Of the total cohort, 76% (15 medical, 4 surgical) exhibited respiratory and/or feeding related symptoms at least once during the period of surveillance (Table 2). The most common symptoms included noisy breathing (68%; 65% medical vs 80% of surgical patients), choking or dysphagia (36%; 25% medical vs 80% surgical), and recurrent emesis (20%; 10% medical vs 60% surgical). Presence of choking/dysphagia (*p* = 0.04), recurrent emesis (*p* = 0.03), and the combination of both digestive symptoms and noisy breathing (*p* = 0.01) were associated with surgical referral. Change in weight z‐scores from newborn visit to last subspecialty follow‐up were similar between medical and surgical patients (*p* = 0.98). During the study period, 15 children (60%; 12 medical, 3 surgical) were discharged from care and 10 (40%) were still followed.

The prevalence of noncritical congenital heart disease was 20%. Arch anatomy determined by transthoracic echocardiogram included 14 cases (56%) with an aberrant left subclavian arterial origin and 11 (44%) with mirror image branching. One child was diagnosed with asthma and one child was diagnosed with obstructive sleep apnea. Extracardiac anomalies were uncommon (< 5%). When performed, neither prenatal nor postnatal genetic testing of the study population revealed abnormalities, including 22q11 deletion syndrome (Table 2).

## 4 Discussion

Pediatric patients with iRAA prenatal diagnosis who present as infants and toddlers with progressive and severe aerodigestive symptoms attributed to a vascular ring are likely to undergo detailed postnatal imaging and surgical evaluation. However, longitudinal clinical data on a cohort of prenatal iRAA patients are not yet available to justify an identical approach for all prenatal iRAA diagnoses. Our study highlights a decade of thoughtful collaboration between fetal cardiologists, pediatric cardiologists, and pediatric pulmonologists following prenatal iRAA diagnoses. We present our institutional management approach (Figure 1) to acknowledge the uncertainty of prenatal vascular ring pathophysiology and provide clinical guidance based on evolution of postnatal symptoms. In studying our postnatal cohort, we observed that most prenatally diagnosed children with iRAA did not develop serious aerodigestive symptoms within their first 2 years of life.

Prenatally a right aortic arch with a left ductus arteriosus represents the anatomic substrate for a vascular ring [7, 8]. However, it does not predict future presence, degree, or progression of postnatal aerodigestive symptoms [16]. Our data demonstrate a variable postnatal clinical course for a cohort of young children with a primary fetal diagnosis of iRAA. Often, even in symptomatic infants, surgical intervention may not be necessary if the infant is thriving and is not experiencing serious developmental compromise related to feeding or sleep. Overall, 80% of infants and toddlers in our study were medically managed in the absence of severe aerodigestive symptoms due to a vascular ring, and growth outcomes were similar between patients referred for surgery and those who were not.

**Figure 3:**
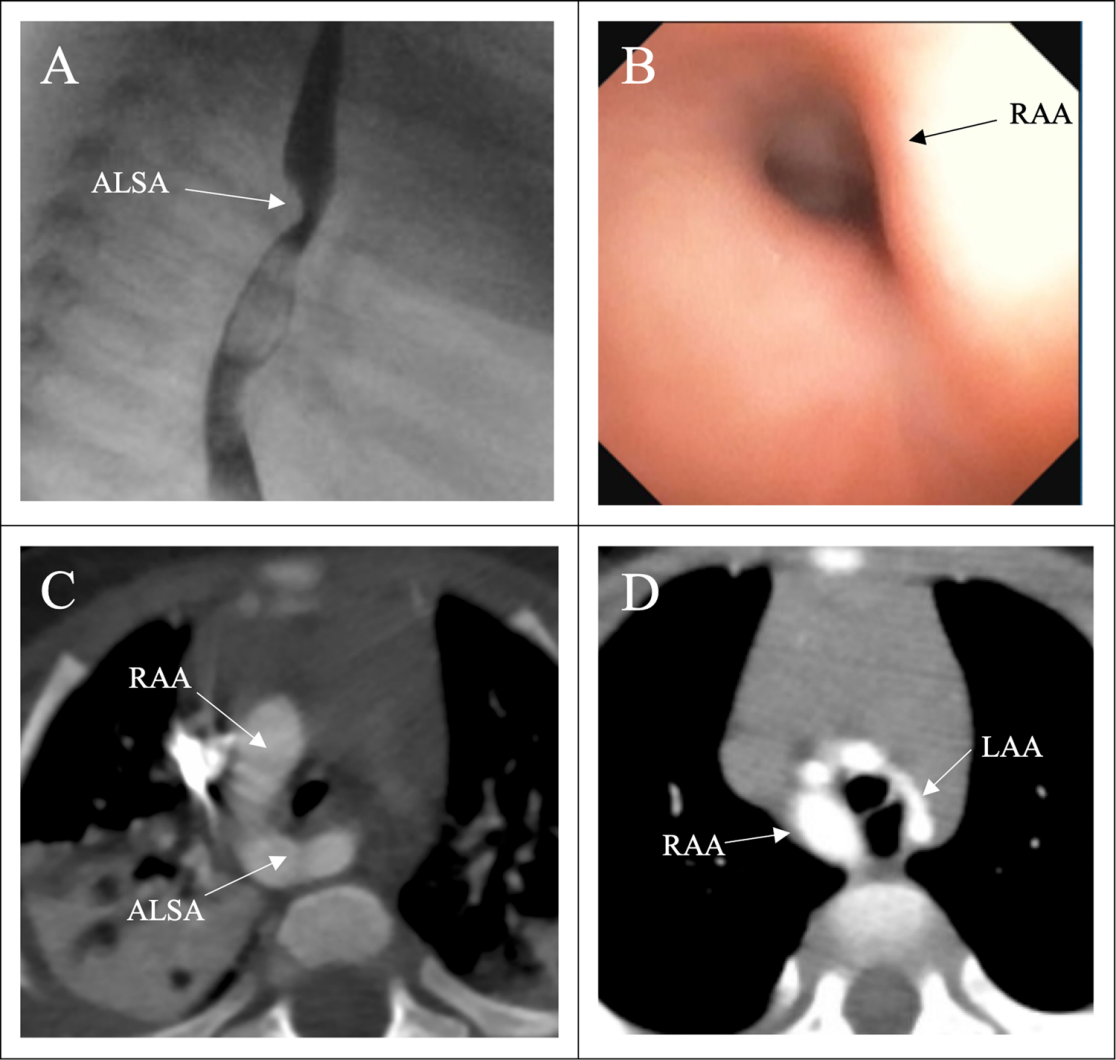
Symptomatic RAA arch evaluation. A. Esophagram demonstrating fixed posterior compression from aberrant left subclavian artery (ALSA). B. Distal tracheal compression by right aortic arch (RAA). C. Chest CT with right aortic arch and aberrant subclavian artery. D. Patient with prenatally diagnosed RAA, found to have right sided dominant double aortic arch and smaller atretic left aortic arch (LAA). [Color figure can be viewed at wileyonlinelibrary.com]

In our review, we identified three common clinical patterns associated with iRAA. First, some patients (5) never developed respiratory or feeding related symptoms. A second group (15 patients) experienced intermittent aerodigestive symptoms that frequently resolved by 24 months of life. They occasionally had progression of their respiratory and/or feeding related symptoms that may not have been a consequence of their vascular anatomy. For example, we followed an infant who had significant upper airway obstruction secondary to adenotonsillar hypertrophy that exacerbated their tracheomalacia. This infant was able to be managed with adenotonsillectomy and improved without the need for vascular surgery. This group also included infants with upper airway or digestive difficulties that were felt to be unrelated to their vascular anatomy. Third, a subset of patients (4) developed progressive symptoms with recurrent lower respiratory infections, increased noisy breathing, or worsening feeding related symptoms, which often coincided with introduction of solid foods. Further radiologic and airway evaluation revealed that the symptoms were most likely related to vascular compression of the trachea and/or esophagus by the right aortic arch and aberrant subclavian artery (Figure 3,A‐C). These infants benefited from surgery. Also within this group, there were instances when echocardiography did not correctly delineate all vascular anatomy. One infant in our cohort, initially described to have mirror image branching, developed progressive symptoms and was ultimately found to have a double aortic arch (predominant right arch with diminutive/nearly interrupted left arch) and managed surgically (Figure 3,D).

Clinical management algorithms for asymptomatic or minimally symptomatic children with iRAA are often center specific, and may empirically recommend more sophisticated imaging, bronchoscopy, and surgery [9, 13, 14, 20, 21]. Prior studies have described that the degree of tracheal compression identified during bronchoscopy does not always correlate with symptom severity and therefore recommend screening bronchoscopy for both symptomatic and asymptomatic patients [12, 21]. Other studies have suggested a risk of impeded tracheal development due to compression from a vascular ring, contributing to recommendations for a lower threshold for invasive testing and surgical intervention [21, 22]. Additionally, the potential long‐term risk of unrepaired iRAA on esophageal motility, neurocognitive development, surgical technique challenges, and quality of life are not well known [17, 23]. Our study contributes important medical data by describing short‐term postnatal outcomes utilizing a clinical algorithm that de‐emphasizes empiric testing, which may be more generalizable for nonsurgical centers.

Over ten years, surgical treatment was recommended for four children (16%) due to progressive aerodigestive symptoms attributed to iRAA vascular ring anatomy, and one child had surgery due to anatomic findings by CT scan. Of note, this patient was referred before the establishment of our institutional collaborative care pathway. Surgeries for children in our pathway were performed between 11 and 14 months of age, and without serious adverse events. Although current surgical interventions are associated with low rates of morbidity, important potential risks remain that are trivial neither to the family nor medical home [9, 17, 18]. Our center followed a cautious approach to surgical referral, considering the complexity of care required to help a child recover from potential recurrent laryngeal nerve injury, chylothorax, or pleural effusion, and the possibility for reoperation [9, 17, 18].

Given the risks of surgery at a young age for mild symptoms compared to unknown long‐term benefits, we deferred early surgical evaluations for 15 patients who developed aerodigestive symptoms and learned that symptoms were either transient or had no clinically significant effect. If we had followed the pathways of other centers, > 80% of our total may have been referred to a surgical center upon developing initial symptoms—even if mild or transiently resolved—and probably would have had empiric testing, possibly increasing exposure to radiation, anesthesia, invasive procedures, and surgery. Instead, we frequently deferred more complex testing and interventions, and during a short period of time learned that many children demonstrated clinical improvement and ultimately were able to be discharged from subspecialty care.

Right arch management recommendations are largely derived from more urban tertiary and quaternary care center data [1, 4, 9, 13, 17, 18, 21]. In more rural areas without surgical services, additional testing and evaluation may be burdensome as families must travel to more specialized facilities. For our patients, these facilities are located out of state, at distances between 150 and 300 miles (estimated 3–5 h travel) from our pediatric center. Given that our data demonstrated that postnatal aerodigestive symptoms are frequently minor and/or transient, and in the context of our regional resources, a more nuanced approach may be warranted when initially counseling families regarding empiric testing or surgery in rural geographies. However, this approach should be cautiously balanced with the importance of regional and centralized surgical expertise often confined to larger centers, where risks and benefits of symptoms, anatomic variants and vascular position may be balanced in relation to surgical options.

Our study addresses the rapid evolution of prenatal diagnostic imaging both in obstetrics and fetal cardiology, which over the past decade has altered the natural history of right arch presentation [1, 2, 3, 4]. Before expanding the cardiac assessment to include the 3VTV, the majority of iRAA diagnoses were likely postnatal, either incidental findings or in the setting of progressive aerodigestive symptoms [1]. Ideally, prenatal testing will continue to evolve, offering more precise anatomic predictors of postnatal symptom severity; yet in the absence of additional congenital heart disease, overall risk stratification is far from perfect. Advancements in prenatal imaging also introduce clinical uncertainty at a far earlier stage—the developing fetus— and may adversely affect parental mental health [24]. Even if lower risk anatomic variation is suspected, congenital findings are more likely to increase diagnostic testing and parental stress during the pregnancy and early childhood years [24, 25]. Our study also demonstrates that nearly 70% (31/45) of right arches in our fetal practice were in the absence of other congenital heart disease, supporting the need for iRAA counseling during pregnancy and updating natural history outcomes relevant to this specific diagnostic context. As fetal diagnosis of iRAA increases, our study offers a collaborative pathway that may support clinicians in accurate and consistent prenatal care counseling, delivery planning, and postnatal care, potentially helping to mitigate parental stress.

## 5 Limitations

Our study was subject to retrospective review limitations with occasionally incomplete or missing data, primarily as families either moved out of the catchment area or did not return to care. Although our median age at last subspecialty follow‐up for medically managed patients was 14 months, which was less than our anticipated observational period of 18 months, presumably these children did not return to follow‐up because they were asymptomatic. For patients who did not return for subspecialty care, their emergency department and primary care visits were systematically assessed for evidence of possible progressive symptoms due to a vascular ring. Although no indicators (surgical records, additional aerodigestive clinical imaging) were found, it is possible that these patients were referred externally for additional management.

While our study included a ten‐year review of a large geographic catchment area, it was not designed as a randomized prospective clinical trial and ultimately yielded a small overall sample size both in medical and surgical groups, which precluded more detailed subgroup analyses. Although there was no clear difference in clinical patterns between children with aberrant left subclavian artery and those with mirror image branching, our study was not powered to detect this anatomic variation as a predictor variable. Our approach limiting the use of cross section imaging may have resulted in underdiagnosis of a double aortic arch, as was present for one patient with a diminutive left aortic arch in addition to the right aortic arch, and should prompt clinicians to consider the expected limitations of fetal and transthoracic echocardiography in this context. Our study did not assess the perceptions of healthcare clinicians or patients regarding the collaborative care pathway. Lastly, while our study offers insights for postnatal iRAA management, it may not be applicable to all anatomic variants, particularly those resulting in more severe initial presentation or at older ages.

Accurate data linkage of maternal prenatal fetal cardiac testing to a postnatal infant record remains a challenge in maternal‐fetal healthcare [6]. Our group has previously described additional infrastructure and informational obstacles for prenatal cardiac testing and postnatal care across a rural geography, methodology to improve accuracy, and standards for congenital heart disease detection [26]. This study sought to improve upon our previously published limitations by utilizing both a retrospective echocardiographic database review and prospective quality improvement maternal/fetal care list. When cross‐referenced, the databases were nearly 90% accurate: All cases identified by echocardiographic review were present by quality improvement review, while four cases identified by quality improvement were not identified by echocardiographic review. This difference was most likely due to an institutional change in echocardiographic software and searchability criteria that occurred in June of 2016. Although it is possible that additional iRAA cases were diagnosed prenatally in our region, our methodology very likely captured the majority of cases, and suggests that prospective quality improvement work may be a valuable adjunct to more traditional imaging database review.

A probable shortcoming of our pathway is consistency of genetic testing and counseling as we did not identify any cases of 22q11 deletion syndrome or other genetic syndromes prenatally or postnatally, which is inconsistent with higher rates in the iRAA literature [4, 16, 18]. Further refinement of our care pathway is expected to involve collaboration with our maternal fetal medicine colleagues to reflect a more consistent genetic testing practice beyond noninvasive prenatal testing. Longitudinal iRAA research is strongly encouraged in larger cohorts to identify and compare longer‐term risks and benefits for both operative and nonoperative management strategies. Quality improvement aims are also encouraged to measure and implement ideal diagnostic testing methodology, assess benefits of parental reassurance when using a standardized and collaborative care algorithm, and evaluate healthcare system costs. Ideally, prenatal care will continue to evolve including expert refinement of anatomic predictors and development of new biomarkers to improve risk stratification of vascular ring pathophysiology.

## 6 Conclusion

Most prenatally diagnosed iRAA children in our cohort did not develop progressive aerodigestive symptoms within their first 2 years of life. Infants more commonly were asymptomatic or had resolution of symptoms and could be managed medically. The presence of isolated digestive symptoms or in combination with respiratory symptoms may prompt greater clinical concern for earlier cross‐section imaging and surgical evaluation. Despite aerodigestive symptoms in the cohort majority, infant weight profiles did not appear to be adversely affected. Standardized postnatal aerodigestive evaluation may assist clinicians with short‐term iRAA risk stratification. Our cardiopulmonary collaborative care pathway offers one symptom‐based framework and supports clinicians to be more judicious about ancillary testing when managing these children in smaller rural and nonsurgical settings.

## Author Contributions

**Jonathan N. Flyer:** conceptualization, investigation, writing – original draft, methodology, validation, visualization, writing – review and editing, formal analysis, project administration, data curation, supervision, resources. **Kelly J. Knight:** investigation, writing – original draft, writing – review and editing, visualization, data curation, project administration. **Erika M. Edwards:** writing – original draft, writing – review and editing, formal analysis, software, validation, methodology. **Caitlin S. Haxel:** writing – review and editing, conceptualization. **Thomas Lahiri:** conceptualization, investigation, writing – original draft, methodology, validation, visualization, writing – review and editing, formal analysis, project administration, data curation, supervision, resources.

## Ethics Statement

All research activities were approved by the University of Vermont Institutional Review Board.

## Conflicts of Interest

The authors declare no conflicts of interest.

## Supporting information

R1 STROBE checklist v4 combined PlosMedicine.

## Data Availability

The authors have nothing to report.
